# The Direct Medical Costs of Late Presentation (<350/mm^3^) of HIV Infection over a 15-Year Period

**DOI:** 10.1155/2012/757135

**Published:** 2011-08-29

**Authors:** Hartmut B. Krentz, M. John Gill

**Affiliations:** ^1^Southern Alberta Clinic, Sheldon M Chumir Health Centre, No. 3223, 1213-4th St SW, Calgary, AB, Canada T2R 0X7; ^2^Department of Medicine, University of Calgary, Calgary, AB, Canada T2N 1N4

## Abstract

We describe the immediate- and longer-term direct medical costs of care for individuals diagnosed with HIV at CD4 counts <350/mm^3^ (“late presenters”). We collected and stratified by initial CD4 count all inpatient, outpatient, and drug costs for all newly diagnosed patients accessing HIV care within Southern Alberta from 1/1/1995 to 1/1/2010. 59% of new patients were late presenters. We found significantly higher costs for late presenters, especially inpatient costs, during the first year after accessing care. Direct medical costs remained almost twice as high for late presenters in subsequent years compared to patients presenting with CD4 counts >350/mm^3^ despite significantly their improved CD4 counts. The sustained high cost for late presenters has implications for recent recommendations for wider routine HIV testing and the earlier initiation of cART. Earlier diagnosis and treatment, while increasing the immediate expenditures within a population, may produce both direct and indirect cost savings in the longer term.

## 1. Introduction

The medical and social aspects of the HIV/AIDS epidemic have been extensively studied since the first cases of AIDS were described in 1981. The medical cost and economic burden to society of the HIV/AIDS epidemic have attracted some but substantially less attention. Early costing studies in the pre-cART (combination antiretroviral therapy) era examined the direct medical costs associated with the morbidity and mortality of AIDS focusing mainly on the costs of hospitalizations [1–7]. These studies also often mentioned that the total economic impact of the epidemic was likely substantially higher than that being measured by direct medical costs when one included the “indirect costs” (i.e., costs not directly attributable to the direct medical cost of HIV/AIDS such as loss of income due to work stoppage) to family members of those living with HIV/AIDS, and the opportunity costs incurred by society from the loss of life from AIDS in a younger, still productive population [8–11]. 

In the pre and early cART eras, costing studies attempted to determine the immediate and lifetime direct costs of HIV disease from the costs associated with various clinically determined stages such as AIDS or CD4+ lymphocyte count. They then predicted the duration that any given patient would be expected to remain in for each one of the stages using a standardized downward trajectory towards eventual death and then generated an estimate of lifetime directs costs for HIV/AIDS [12–17]. This methodology was viewed as generally being valid as few, if any, effective treatments were available to slow disease progression. 

With the arrival in 1996 and subsequent widespread implementation of cART, the HIV epidemic changed significantly. Morbidity and mortality from HIV decreased increasing patients' health, survival, and overall lifespan [18, 19]. The economic burden measured by direct medical costs has shifted from inpatient costs (i.e., hospitalizations) to outpatient costs primarily reflected as the cost of the ARV (antiretroviral) drugs, outpatient visits, and laboratory tests [20–26]. The success of cART is likely even to be greater than measured in direct costs as it has allowed most patients to live not only longer and healthier lives, but to maintain the individual's productivity thereby decreasing the indirect and opportunity costs to family members and to society in general.

Costing of the HIV epidemic has become far more complex in the cART era as the disease trajectory is no longer a predictable decline. Many patients experience a CD4 increase after starting cART, some maintain stable CD4 counts while on cART, and some remain with low CD4 counts but suppressed viremia [27–29]. As such, it has become increasingly difficult to determine how long any patient would remain in a particular disease “stage” using the CD4 count as the stage marker. Costs within any CD4 stratum may vary widely depending upon the mix of patients with untreated disease or with disease recovering on cART. This heterogeneity makes this methodology no longer easily usable on large populations [30–32]. 

Individuals infected with HIV may also access care for the first time at different stages of their HIV infection (based on their CD4+ lymphocyte counts). These stages at presentation carry both health and economic implications. The term “*late presenters*” was originally used to indicate a person who initiates HIV care at a “late” stage of their disease or with a lower CD4 count (i.e., <200/mm^3^) indicating poorer health and poorer health outcomes [33–39]. These studies indicated that these “late” patients had not only higher mortality and morbidity than patients presenting “early” but also incurred substantially more direct medical costs [17, 31, 32]. With cART, however, mortality and morbidity rates as well as costs and the distribution of costs have changed for late presenters. It has also been proposed that the term “late presenter” be modified [40] to reflect “late for care” with the CD4 threshold moving to CD4 <350/mm^3^ and the term “advanced disease” introduced to reflect CD4 <200/mm^3^. These adjustments will make comparisons between historical and current studies difficult unless the definition of a “late presenter” is clearly presented. 

Using our costing database, we examined in this paper the cost of late presentation (CD4 < 350/mm^3^) over a 15-year period describing past and current trends. We determined the cost of care of both late and “early” presenters (i.e., patients who access initial HIV care with CD4 counts >350/mm^3^) over time comparing costs after accessing HIV care. We discuss the impact of late presentation on current recommendations for more widespread and routine HIV screening and testing, and on the proposed “test and treat” strategies under discussion. Late presentation has not only clinical and public health implications within the HIV epidemic but also has financial and costing implications.

## 2. Methods

The Southern Alberta Clinic Cohort (SAC) includes all HIV-infected patients receiving HIV care and living within southern Alberta, Canada. Patients are automatically included in the cohort when they initiate HIV care within a centralised outpatient program. SAC provides exclusive, comprehensive interdisciplinary care to all HIV patients living in southern Alberta including pharmaceuticals, outpatients, and laboratory tests. All individuals testing positive for HIV are referred to SAC located in Calgary, Alberta. Over 90% of patients reside within the immediate Calgary region. Inpatient services are provided in one of 3 local hospitals. 

Administrative data including demographic, clinical characteristics as well as the direct cost of care are collected on all individuals on a routine basis during every clinical contact. Use of this administrative data was approved by the University Conjoint Medical committee on medical bioethics.

We include all newly infected HIV individuals diagnosed within the region who accessed their initial HIV treatment at SAC (“locally diagnosed patients”). Individuals who were diagnosed elsewhere were included if they were initiated care within 6 months of their diagnosis and had not accessed HIV elsewhere prior to their 1st SAC visit. We include all individuals initiating care between 1 Jan 1995 and 1 Jan 2010. To be included, patients must have had at least one regular clinic visit. Patients were followed until they moved, were lost to followed, died or until 1 April 2010.

We use the definition of “late presenters” as those patients who initiated care with a CD4 count <350/mm^3^ although we also subdivide this group by CD4 count > or < than 200/mm^3^ for comparisons with earlier uses of the term “late presenters.” We collected the patient's gender, age at clinic visit, risk factor (MSM, MSW, IVDU, other) and self-reported ethnicity (Caucasian/non-Caucasian) at the initial visit. We recorded the patient's initial CD4 count taken within 30 days of the initial visit and any recorded AIDS defining condition at diagnosis. 

The Southern Alberta Clinic Cohort has been continuously tracking the direct cost of care for all HIV-infected patients followed at the regionalized Southern Alberta Clinic. SAC established a “costing search engine” that routinely captures all the direct costs of care including ARV (antiretroviral) and non-ARV drug costs, all outpatient clinic visits including laboratory texts and referrals to non-HIV specialists, and the cost of inpatient (i.e., hospitalizations) visits for both HIV and non-HIV-related admissions. Costs are collected per patient, per demographic population, or per a number of other variables including the CD4 status of the individual patient. 

For this study, the direct costs of care were collected between 1/1/1995 and 12/31/2009. Costs were collected from the original costing source or agency using a methodology previously described [16]. Briefly, we collected the direct costs of drugs (antiretroviral (ARV) and nonantiretroviral drugs), outpatient clinical care (including physician and laboratory costs), and inpatient (hospital) care. ARV and non-ARV drug costs, lab utilization, and outpatient care costs were derived directly from the SAC pharmacy, Calgary Laboratory Services, and the SAC-costing database whereas inpatient costs (i.e., unit service costs) were supplied by the regional health service providers. The unit costs used are market values charged to the regional payer (Alberta Health Services). All costs were obtained directly from the costing agencies and reported in Canadian dollars adjusted for inflation to 2009. 

Annual costs for patients who initiate HIV care at SAC are reported from the date of initiating year to December 31st of that particular year. Costs are then adjusted as mean cost per patient per month (PPPM) in 2009 Cdn$ over the time followed in that year, and cumulatively for patients initiating care ±350/mm^3^. The annual cost for “late presenters” is reported as a proportion of all costs for newly diagnosed HIV patients accessing care for the first time. Long-term or “lifetime” costs are determined from the date of initiating HIV care to the date they moved, were LTFU (lost to followup), died, or 4/1/2010 and, are reported as mean PPPM or PPPY (per patient per year) costs. 

Health care utilization data is based on number of clinic visits, laboratory tests, visits to HIV, and non-HIV physicians (i.e., outpatient visits), and the number of hospital admissions (inpatient visits/length of stay (LOS)). Administrative data were obtained directly from the SAC database and hospitalization admission records. Visits for physicians for non-HIV related conditions were self reported by the patients and may be underreported. Clinical protocols on recommended frequency of clinic visits, ART options, and laboratory testing algorithms for patients remained stable during the study period.

We compare the PPPM cost of care for late presenters initiating care at SAC to that of early presenters over the same time period and under the same clinical protocols. We provide descriptive statistics (i.e., mean, standard deviations, medians) to describe the data. We use Student *t*-tests for normally distributed data and Mann Whitney *U*-test for non normally distributed variables to compare the populations. Chi-square tests were used to compare proportions. *P* < .05 was set for the level of significance.

## 3. Results

**Figure 1:**
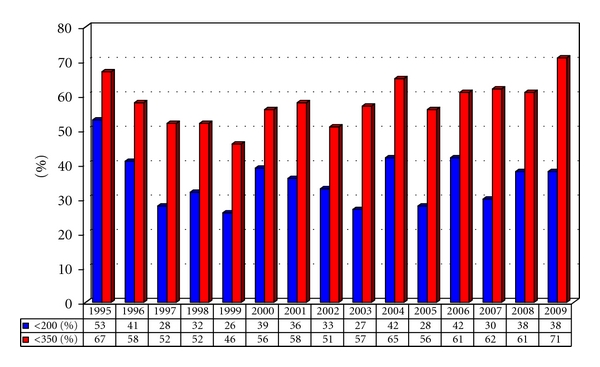
Proportions of newly diagnosed HIV patients accessing care with CD4 counts <200/mm^3^ (“advanced disease”) and/or <350/mm^3^ (“late presenters”).

The demographic and clinical characteristics of late presenters are listed in Table 1. Between 1995 and 2010, 59% of all locally diagnosed patients initiated care with a CD4 <350/mm^3^ (36% with CD4 counts <200/mm^3^) as shown in Figure 1. We found a change in the demographics of late presenters during this period. In 1995, 89% were male, 66% were MSM (men who have sex with men), and 79% were Caucasian; in 2009, 73% were male, 45% MSM (43% were MSW), and 48% were Caucasian. The median CD4 count for late presenters was 149/mm^3^ (IQR [47-253]); 26% of late presenters had an AIDS defining condition at time of accessing care. 9.6% of late presenters died within 60 months of accessing care. 

**Figure 2:**
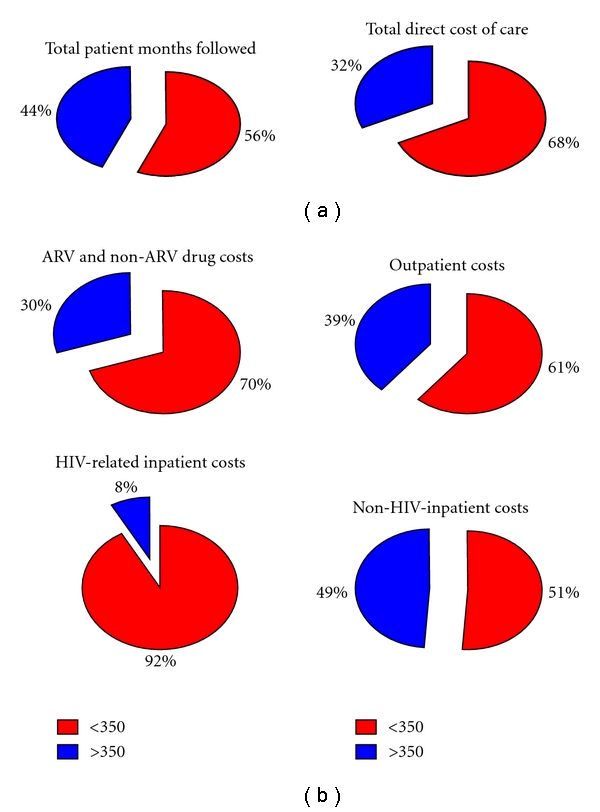
(a) Proportional costs of direct medical care with for newly diagnosed HIV patients accessing care from 1995 to 2010, (b) catergorized by cost category.

Over the past 15 years, locally diagnosed “late presenters” account for 56% of the total patient months followed at SAC compared to 44% for early presenters (>350/mm^3^); however, they account for >68% of all costs (Figure 2(a)). Overall, 70% of all drug costs (69% of ARV drug costs, 84% of all non-ARV drug costs), 61% of all outpatient costs, and 64% of hospital costs (92% of HIV-related hospital costs and 51% of non-HIV-related hospital costs) were attributable to late presenters (Figure 2(b)). 

**Figure 3:**
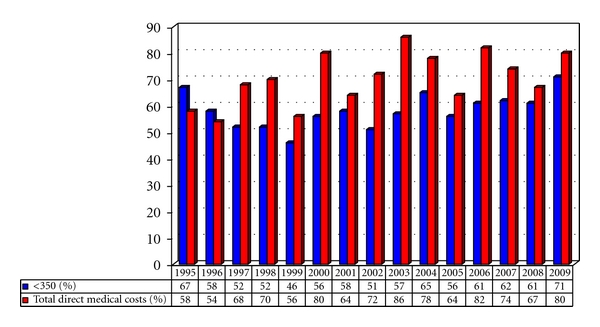
Proportions of the total direct medical costs incurred by “late presenters” (<350/mm^3^) as a percentage of all direct medical costs for newly diagnosed HIV patients accessing care.

The proportional annual cost of care for late presenters versus early presenters for the year the person was diagnosed is presented in Figure 3. With the increased use of cART and with the trend at initiating cART at higher CD4 counts, we found that the proportional costs for late presenters increased substantially over the past 15 years—from 60% between 1995 and 1999 to over 75% between 2000 and 2009. Inpatient costs account for nearly two thirds (i.e., 64%) of all the costs incurred during the first year after accessing HIV for late presenters. 

**Figure 4:**
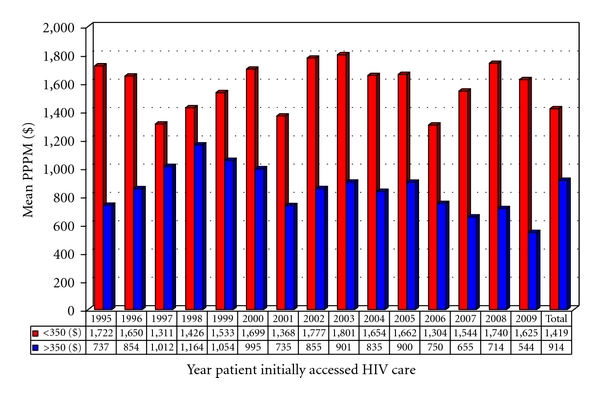
Mean cumulative PPPM (per patient per month) total cost of care for HIV patients accessing care in the year listed and followed until the patient moved, died, or 12/31/2009 in 2009 Cdn$.

Patients who present late continue to cost more despite a recovery in their health in subsequent years beyond their initial year of diagnosis (Figure 4). Overall, late presenters cost a mean of $1419 ± $378 per month ($17,028 ± $5,031 per year) compared to $914 ± $452 per month ($10,968 ± $5,677 per year) for early presenters. Although there is yearly variation, mean PPPM costs remain substantially higher every year throughout the past 15 years. This substantial difference is also seen for patients who have been continuously followed at SAC from initial time of access care to the end of 2009. The mean initial CD4 count for late presenters was 122/mm^3^ at first visit and 437/mm^3^ at their latest CD4 count in 2009 compared to 470/mm^3^ and 566/mm^3^, respectively, for early presenters yet mean PPPM cost for these “late presenters” for the year 2009 remained almost twice as high (i.e., $1477 ± $402 versus $896 ± $366) despite significant improvements in CD4 counts.

## 4. Discussion

We have documented that over the past 15 years the direct cost of care has remained significantly higher (>50%) for HIV-infected patients who present with a CD4 count <350/mm^3^. These costs are not exclusively derived from the use of cART but reflect all direct medical costs. We have also shown that these increased direct costs are sustained beyond the initial year of care after presentation and persist despite CD4-rebound and -improved health. Late presenters continue after presentation to use not just more cART and outpatient care but more inpatient care, and, more non-ARV drugs. These costs may not only reflect lifelong legacy costs of the residual morbidities from some AIDS conditions but also may reflect the costs of complex social and medical issues that contributed to late presentation (e.g. denial, psychiatric illness, substance use). The rate of hospital admissions in late presenters is higher for both HIV and non-HIV-related conditions both at initial presentation and in subsequent years suggestive of the importance of legacy morbidity and comorbidities. Fleishman et al. [32] also documented substantially higher continuing direct medical costs in the United States for late entrants to HIV care even after 7 to 8 years in care. They state that earlier entry into HIV care at relatively less costly disease stages could reduce aggregate expenditures. Our findings concur. 

The center for disease control [41] recommended in 2006 more widespread and routine HIV testing as a means to detect more of the 20% to 33% of individuals who currently unaware they are HIV infected. The findings from this and other similar costing studies, carry implications with regard to assessing the economic impact of these recommendations as well as for the associated increased cART use for patients successfully engaged in HIV care. 

It is anticipated that a substantial number of individuals identifying earlier with lower CD4 counts through this wider testing process will successfully engage in care, receive cART, decrease their infectivity (and the rate of secondary infections), and improve their own health. The costs of wider testing and the increased use of cART may be defrayed by decreasing the substantial and sustained direct medical costs from later presentation, the indirect costs to family from an avoidable illness (i.e., presentation with HIV/AIDS), and the opportunity costs to society by minimising lost productivity and reducing secondary infections [42, 43].

It is argued that the largest societal cost impact of earlier and more widespread detection of HIV and engagement to care will be the public health effect of “infections prevented.” Proponents of the “test and treat” strategies for HIV prevention stated that expanded testing and earlier treatment could markedly decrease ongoing HIV infection and, in time, stem the HIV epidemic [44–46]. Those on treatment will have decreased viral loads and be less infectious and, in principle, should decrease to some degree new infections. The precise reduction in new infections from such a strategy within a population remains highly speculative along with its predicted savings both in actual costs and in reduced HIV transmission [47–49]. 

On a population level beyond the costs of wider testing, the cost of HIV care will increase as the number of individuals diagnosed with HIV and on treatment will increase. We have previously shown that the overall cost of care for HIV-infected individuals will increase within a population as more individuals are detected and begin to access HIV care and ARV drugs [50]. We estimated that wider screening and initiated HIV care would increase HIV costs by 21 to 28% if over half of the currently unidentified individuals with HIV infection were identified and accessed care. However, on an individual basis, patients who access care at a higher CD4 count have much lower cost PPPM over the course of their condition compared to individuals who access care at lower CD4 counts. We have shown how costs remain high over at least 7 years or more of followup despite improved health. As both early and late presenters now live longer and require sustained treatment and management of their condition, the difference in the cost PPPM between these groups will continue to be disproportional. 

Our study, while comprehensive, does have limitations. Many factors including the ease and availability of accessing care, the composition of the HIV community, the location of the HIV care site or sites, the cost of direct or indirect health care within the community, the use and preference by ARV's by health care providers, and other aspects of care delivery may influence mean cost PPPM over time and between geographic locations. Collection of costing data itself may increase or decrease actual costing estimates. We have attempted to reduce many of the factors by concentrating on only those patients diagnosed and accessing care at a centralized care center within a defined geographic population over the course of 15 years in which there was a continuity in clinic protocols and management philosophy driven by international guidelines. Costing collection and the methodologies applied have remained the same over the study period. Although the actual costs of ARV medicine, outpatient and inpatient care may be higher or lower than other centers due to differences in health care systems across and inside any country, the proportional differences we identified are remarkably similar to those reported by others in costing studies, and, thus, the analysis and discussion should be widely applicable. Our study also only reports on costs in a developed country and as such is not directly relevant to costing studies in developing nations where clinical, demographic, and economic issues are significantly different [51–54], at least to a degree. The underlying aspects and costing principles presented in our study can be applied to other situations albeit with differing cost estimates. 

 Two other considerations will need to be addressed in future costing studies. More and more ARV drugs will be coming off patent in the near future and will be available in generic form. This most likely will directly or indirectly reduce the cost of ARV drugs and regimens and should reduce long-term costs of care for HIV-infected individuals. How much and how quickly these costs change will increasingly make future costing projections less precise. Another important aspect to be addressed is the cost savings in indirect costs and opportunity costs from cART therapy. Improving the health of HIV patients and increasing their longevity not only is beneficial to the patient's health but its major impact is likely in minimising indirect cost to patients family for caring and in reducing opportunity cost to society form lost productivity. Future studies need to explore such issues to further measure the economic impact of early identification and treatment with cART.

## 5. Conclusion

HIV/AIDS has been and continues to be an expensive disease to manage. Early detection and treatment of the HIV infection has been shown to produce very positive clinical and public health effects; however, at the same time, direct medical care costs increase as patients initiate cART earlier and over longer-time periods. Increased initial costs can be defrayed over time by more stable and lower costs of care as health improves. Many costly hospitalizations may be avoided with proper disease management. Earlier detection and access to HIV care may also reduce indirect costs as patients maintain productive lifestyles to the best of their abilities thus also reducing societal costs. The high initial and sustained costs of late presentation in HIV disease is a factor in discussions on more widespread testing and treatment of HIV disease.

## Figures and Tables

**Table 1:** Demographic and clinical characteristics of patients followed within the Southern Alberta Clinic Cohort from 1995 to 2009 (selected years only) accessing initial HIV care with CD4 counts <350/mm^3^ (“late presenters”).

	1995	2000	2005	2009
Total no. of late presenters (%)	47 (67)	34 (56)	45 (56)	67 (71)

Male (%)	42 (89)	39 (87)	36 (80)	49 (73)

Median age (yrs)	30	32	33	34
[IQR]	[26–37]	[27–39]	[27–40]	[28–41]

Risk factor				
MSM (%)	31 (66)	20 (58)	24 (54)	30 (45)
Heterosexual	8 (17)	5 (16)	11 (24)	29 (43)
IVDU	7 (15)	7 (22)	9 (20)	7 (10)
Other	1 (2)	2 (4)	1 (2)	1 (2)

Caucasian (%)	37 (79)	24 (70)	25 (55)	32 (48)

Median initial CD4	123	55	159	193
[IQR]	[36–211]	[10–214]	[80–261]	[63–263]
